# Effects of rhBMP-2 on Sandblasted and Acid Etched Titanium Implant Surfaces on Bone Regeneration and Osseointegration: Spilt-Mouth Designed Pilot Study

**DOI:** 10.1155/2015/459393

**Published:** 2015-10-04

**Authors:** Nam-Ho Kim, So-Hyoun Lee, Jae-Jun Ryu, Kyung-Hee Choi, Jung-Bo Huh

**Affiliations:** ^1^Department of Dentistry, College of Medicine, Korea University, Anam-Dong 5-Ga, Seongbuk-gu 136-705, Republic of Korea; ^2^Department of Prosthodontics, Dental Research Institute, Biomedical Research Institute, School of Dentistry, Pusan National University, Yangsan 676-870, Republic of Korea; ^3^Research Development Institute, Cowellmedi Co., Ltd., Busan 617-801, Republic of Korea

## Abstract

This study was conducted to evaluate effects of rhBMP-2 applied at different concentrations to sandblasted and acid etched (SLA) implants on osseointegration and bone regeneration in a bone defect of beagle dogs as pilot study using split-mouth design. *Methods*. For experimental groups, SLA implants were coated with different concentrations of rhBMP-2 (0.1, 0.5, and 1 mg/mL). After assessment of surface characteristics and rhBMP-2 releasing profile, the experimental groups and untreated control groups (*n* = 6 in each group, two animals in each group) were placed in split-mouth designed animal models with buccal open defect. At 8 weeks after implant placement, implant stability quotients (ISQ) values were recorded and vertical bone height (VBH, mm), bone-to-implant contact ratio (BIC, %), and bone volume (BV, %) in the upper 3 mm defect areas were measured. *Results*. The ISQ values were highest in the 1.0 group. Mean values of VBH (mm), BIC (%), and BV (%) were greater in the 0.5 mg/mL and 1.0 mg/mL groups than those in 0.1 and control groups in buccal defect areas. *Conclusion*. In the open defect area surrounding the SLA implant, coating with 0.5 and 1.0 mg/mL concentrations of rhBMP-2 was more effective, compared with untreated group, in promoting bone regeneration and osseointegration.

## 1. Introduction

The main goal of a dental implant for patients and dentists is to reduce the healing period from implant fixture placement to prosthesis mounting while achieving successful functional gains after surgery. The most important contributor to reducing healing time is the achievement of osseointegration during the early stage and its subsequent maintenance [1]. The implant surface is an important contributor to successful osseointegration and it clearly plays a key role in patients with inadequate bone quality and quantity [2]. Recent studies on implant surfaces have focused on topography and changes in chemical properties that enhance implant/bone integration [3, 4]. Surface treatment methods such as titanium plasma-spraying, grit-blasting, acid etching, anodization, and coating with inorganic calcium phosphate have been suggested to reduce healing times, increase osseointegration, and improve augmentation of surrounding bone [5, 6]. The treatments of resorbable blast media (RBM), sandblasting with large grit followed by acid etching (SLA), and anodic spark deposition (ASD) are widely used in clinical field and have been shown to provide effective osseointegration in many studies [7–10].

Recently, attempts have been made to improve the success rate of implantation in bone defect site by employing biomimetic surface treatments [11]. Biomolecules, such as Arg-Gly-Asp (RGD) peptide [12], extracellular protein (collagen) [13], and growth factor [14], are used to promote bone healing and osseointegration. Bone morphogenetic proteins (BMPs), which belong to the transformation growth factor beta superfamily, induce the differentiation of osteoblasts from mesenchymal stem cells as well as the biosynthesis of bone matrix and accelerate ossification by controlling essential factors of the bone induction cascade [15, 16]. In particular, BMP-2 has been reported to have high osteogenic activity [17], and its ability to enhance bone regeneration has been demonstrated in previous studies, which include studies on augmentation of the maxillary sinus floor [18], bone formation and reosseointegration in peri-implant defects [19], local alveolar ridge preservation or augmentation [20], and periodontal repair [21]. In a recent study, the affinity of BMP for titanium was confirmed, and thus titanium implants can be considered potential BMP carriers [22–24]. Huh et al. [25] reported effective implant stability and alveolar bone formation as determined using implant stability quotient (ISQ) values and histological observations at 8 weeks after placing an anodized implant coated with* E. coli*-derived rhBMP-2 (ErhBMP-2) in a 2.5 mm supra-alveolar vertical bone defect animal model.

However, several studies have failed to validate the effectiveness of rhBMP-2* in vivo*, presumably due to its short half-life and lack of an optimal concentration, when used without other growth factors [26–28]. Since anodized implants coated with rhBMP-2 of 0.75 or 1.5 mg/mL induced clinically relevant local bone formation, including vertical augmentation and osseointegration of alveolar bone, these concentrations were deemed appropriate for the surface treatment of TPO implants [29, 30]. SLA surface treatment is widely used commercially to improve the biocompatibilities of titanium implants and has been shown to increase bone apposition during the initial osseointegration period [7] and to increase bone-to-implant contact and removal torque values as compared with titanium-plasma-sprayed (TPS) surfaces [8]. In addition, the SLA surface treatment was reported with 97% success rate on clinical case as loaded after healing period of 6 weeks [31]. Lee et al. [32] examined three different implant surfaces (SLA, RBM, and MgO) treated with rhBMP-2 at 1.5 mg/mL in a rabbit model. They found out that the surface rhBMP-2 amounts were surface dependent and that the valid amounts were greater on SLA surfaces. However, histomorphometric analysis showed rhBMP-2/SLA implants had the lowest bone-to-implant contact at cortical bone and around implant threads. Although surface loadings of rhBMP-2 increased with SLA implant irregularity and roughness, a treatment with rhBMP-2 at 1.5 mg/mL failed to achieve appropriate osseointegration and bone regeneration. The optimized rhBMP-2 concentration is the key factor in order to use SLA implants as effective rhBMP-2 carriers, but it is still unknown.

Therefore, the aims of the present study were, first, to evaluate effects of rhBMP-2, applied at 3 different concentrations [24, 33] to SLA implants, on osseointegration and bone regeneration in a bone defect beagle dog and, lastly, to identify an optimal rhBMP-2 concentration.

## 2. Methods

### 2.1. Preparation of SLA Implant Coated with rhBMP-2

Twenty-four implants (7.0 mm long and 3.5 mm in diameter; Cowellmedi Co., Busan, Korea) were made of pure titanium and designed with a straight section on their upper 3 mm and broad threads on their lower 4 mm. The implant surface was sandblasted with large grit and acid etched (SLA, Cowellmedi Co., Busan, Korea). To coat implants with rhBMP-2 (Cowellmedi Co., Busan, Korea), each implant was immersed 3 times in a protein solution (0.1, 0.5, and 1.0 mg/mL) up to the beginning of the straight portion, frozen, and dried under sterile conditions (at −40°C) and then vacuum dried at maximum 20°C. Control SLA implants were not treated. Each group contained 6 implants.

### 2.2. Surface Characterization by Scanning Electron Microscopy (SEM)

Implant surface morphologies were investigated using a SEM (S2300, Hitachi, Japan). Substrates sputter coated (Elko IB, Japan) with gold and the SEM were operated at 15 kV.

### 2.3. Determination of Amounts of rhBMP-2 on SLA Surface

Three random implants were selected from each experimental group, and the amount of surface BMP-2 was determined by using a human BMP-2 standard ELISA development kit (900-K255, Pep Rotech, Rocky Hill, NJ, USA). All groups were diluted to 0, 62.5, 125, 250, 500, 1000, 2000, and 4000 pg/mL using 5x diluents solution according to the manufacturer's instruction. 100 *μ*L of each prepared group was dispensed into antibody coated plate, incubated at room temperature for 2 hours, and then washed 4 times. After dispensing 100 *μ*L detection antibody into each well, the wells were allowed to react at room temperature for 2 hours and then washed 4 times. After dispensing 100 *μ*L SA-HRP (streptavidin horseradish peroxidase) solution and reacting for 30 minutes, the wells were washed. After the reaction at which they were dispensed with 100 *μ*L toluidine and methylene blue solution for 20 minutes, with the light blocked, absorbance was measured at 450 nm using a microplate reader (VersaMax, Molecular Devices, Sunnyvale, CA, USA).

### 2.4. Determination of the Amount of rhBMP-2 Released from SLA Surface

Samples were immersed in 50 mL conical tubes containing 1 mL of PBS buffer (pH 7.4). Tubes were then gently shaken at 100 rpm at 37.8°C to evaluate the amount of rhBMP-2 released from SLA surface. The supernatants were collected and replaced with fresh PBS at predetermined times (1 h, 3 h, 6 h, and 12 h and 2, 4, and 30 days). All samples were stored at −20°C until being required for analysis. The amounts of rhBMP-2 released from surfaces were determined using an ELISA kit and a microplate reader (Bio-Rad, Hercules, CA, USA) at 450 nm. Cumulative releases of rhBMP-2 are expressed as percentages of initial loadings.

### 2.5. Experiment Animals

This study was conducted with the approval of the Ethics Committee on Animal Experimentation of Chunnam National University (CNU IACUC-TB- 2013-10). Four 3-year-old beagle dogs, approximately 13–15 kg in weight, were used, and all animals were acclimatized for 2 weeks. During the experiment, the dogs were fed with a soft-food diet and had free access to water.

### 2.6. Surgery for Tooth Extraction

During the first surgery, premolars and first molars of lower jaws were extracted. Animals were preanesthetized with atropine sulfate induction (0.05 mg/kg IM; Dai Han Pharm Co., Seoul, Korea) and maintained by isoflurane (Choongwae Co., Seoul, Korea) gas anesthesia. Lidocaine (1 mL; Yu-Han Co., Gunpo, Korea) with 1 : 100,000 epinephrine was injected into the mucosa of surgical sites. Lower premolars and first molars were then separated into two pieces, mesial and distal roots. Teeth were extracted carefully to avoid damage in extraction sites, which were subsequently sutured with 4-0 nylon (Mersilk, Ethicon Co., Livingston, UK). The sites were allowed to heal for 2 months.

### 2.7. Surgery for Implantation

**Figure 1:**
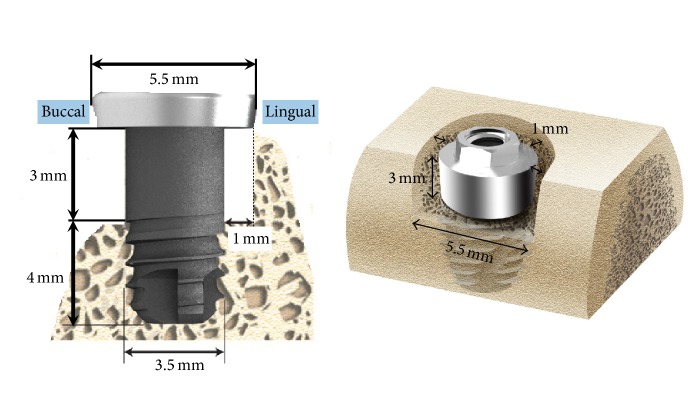
Experimental implant design and schematic diagram of the buccal open defect and mesial, distal, and lingual 1 mm defect models. Twenty-four implants (7.0 mm long and 3.5 mm in diameter; Cowellmedi Co., Busan, Korea) were made of pure titanium and were designed with a straight section on their upper 3 mm and broad threads on their lower 4 mm. A 5.5 mm diameter cover screw mounted on the implant.

**Figure 2:**
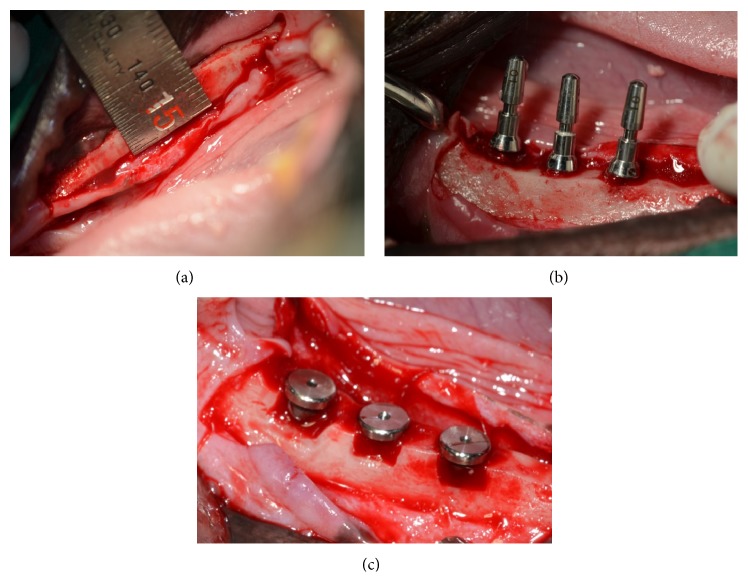
Surgical procedures. (a) Alveolar bone was flattened. (b) 5.5 mm width peri-implant defects were created using a trephine bur (Dentech, Tokyo). (c) The implant was placed within its prepared site, and peri-implant defect areas were covered using a 5.5 mm diameter cover screw.

Implant surgery was performed 2 months after tooth extraction, that is, after complete healing had been achieved. Local and general anesthesia were performed the same as described above for tooth extraction. Alveolar ridges were trimmed by about 1.5 mm to produce a flat ridge before implant insertion. The defects produced were 3 mm deep and 5.5 mm wide. This model had a buccal open defect and a 1 mm defect area around the 3 mm high upper implant portion (Figure 1). Four animals received implants coated with rhBMP-2 at 0.1 or 0.5 or 1.0 mg/mL or uncoated controls in contralateral jaw quadrants using split-mouth design. Treatments of left and right jaw quadrants were randomized. To place implants at the same location at both sides, exposed bone was marked using a ruler at each implant placement site. Each upper implant area coated with rhBMP-2 was fixed with a 5.5 mm diameter cover screw, which was large enough to cover the defect area and minimize rhBMP-2 loss (Figure 2). The mucoperiosteal flaps were advanced, adapted, and sutured while the implants were left submerged.

### 2.8. Animal Postoperative Care and Sacrifice

Broad spectrum antibiotics (penicillin G, procaine, and penicillin G benzathine) were administered immediately after implantation and again 48 hours later by intramuscular injection (1 mL/5 kg). Plaque was controlled by daily tooth brushing with 2% chlorhexidine gluconate until the completion of the study. The mucosal health, maintenance of suture line closure, edema, and evidence of tissue necrosis or infection at experiment sites were observed daily until suture removal at 1 week after implant placement. The animals were given a soft diet for 2 weeks and then a conventional regular diet. Mineralized tissue was labeled and the time courses of new bone formation and mineralization were assessed using the polychrome sequential fluorescent labeling method. Calcein (20 mg/kg, Sigma, USA) was intravenously injected 2 weeks after implantation, and Alizarin Red S (30 mg/kg, Sigma, USA) was injected i.v. 6 weeks after implantation. At 8 weeks, animals were anesthetized and euthanized by intravenously injecting concentrated sodium pentobarbital (Euthasol, Delmarva Laboratories Inc., Midlothian, VA, USA). Block sections including implants, alveolar bone, and surrounding mucosa were then collected.

### 2.9. Assessment of Implant Stability

Implant stability quotients (ISQ) were calculated for mandibular implants using Osstell Mentor (Integration Diagnostic Ltd., Göteborg, Sweden) immediately after implantation and at 8 weeks later. ISQ values were recorded 5 times for each implant, but minimum and maximum values were excluded to calculate means and standard deviations (SDs).

### 2.10. Fluorescence Analysis

Dynamic bone mineral deposition was assessed by sequential fluorochrome labeling. The histomorphometric analysis of fluorochrome labeling was performed by confocal laser scanning microscopy (Leica, TCS Sp2 AOBS, Germany). The excitation and emission wavelengths of the chelating fluorochromes were 494/517 nm and 530–560/580 nm for Calcein (green, at 2 weeks) and Alizarin Red S (red, at 6 weeks), respectively. Fluorescent quantification of Calcein and Alizarin Red S was used to determine the new bone formation and mineralization. Mineralization levels were determined at different time points.

### 2.11. Histomorphometric Analysis

The specimens were fixed in neutral buffered formalin (Sigma Aldrich, St. Louis, MO, USA) for 2 weeks and dehydrated in an ascending ethanol gradient (70%, 80%, 90%, and 100%). Dehydrated specimens were embedded in Technovit 7200 resin (Heraeus KULZER, South Bend, IN, USA). Blocks of polymerized specimens were sectioned longitudinally at the center of each implant using an EXAKT diamond cutter (KULZER EXAKT 300, EXAKT, Norderstedt, Germany). Final sections (30 *μ*m) were prepared from the initial 400 *μ*m sections by grinding using an EXAKT grinding machine (KULZER EXAKT 400 CS, EXAKT, Norderstedt, Germany). The specimens were stained with hematoxylin and eosin, and images were captured using a light microscope (Olympus BX, Tokyo, Japan) equipped with a computer and a CCD camera [Polaroid DMC2 digital microscope camera (Polaroid Corporation, Cambridge, MA, USA)]. Three parameters, that is, vertical bone height (VBH, mm), bone-to-implant contact ratio (BIC, %), and bone volume (BV, %) in the upper 3 mm straight implant portion in the buccal open defect and lingual peri-implant defect areas, were measured using SPOT Software V4.0 (Diagnostic Instruments, Inc., Sterling Height, MI, USA). Specimen images were captured at a magnification of ×12.5, histomorphometric analysis was conducted at a magnification of ×40, and a precise assessment of BIC was done at ×100.

## 3. Results

### 3.1. Surface Characteristics

**Figure 3:**
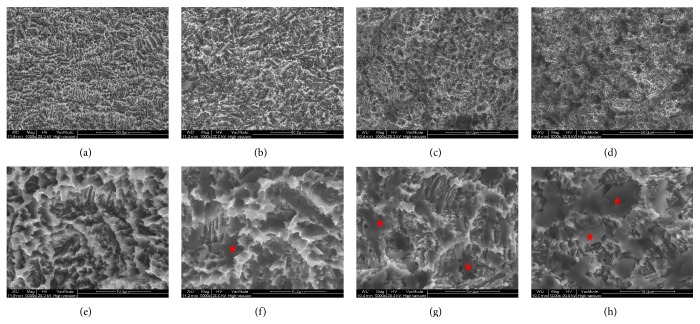
SEM images of each group. (a, e) The control group, (b, f) the 0.1 group, (c, g) the 0.5 group, and (d, h) the 1.0 group. Asterisk: freeze dried rhBMP-2: (a, b, c, d) ×1000, (e, f, g, h) ×5000.

SEM images of SLA implants uncoated (the control group) or coated with rhBMP-2 (the 0.1, 0.5, and 1.0 groups) are shown in Figures 3(a)–3(h). The images show that the rhBMP-2 layer was formed on roughened surfaces. The amounts of surface rhBMP-2 in the 0.1, 0.5, and 1.0 groups were 0.965 ± 0.049, 3.212 ± 0.806, and 9.354 ± 1.270 *μ*g/ea., respectively.

### 3.2. rhBMP-2 Release Test

**Figure 4:**
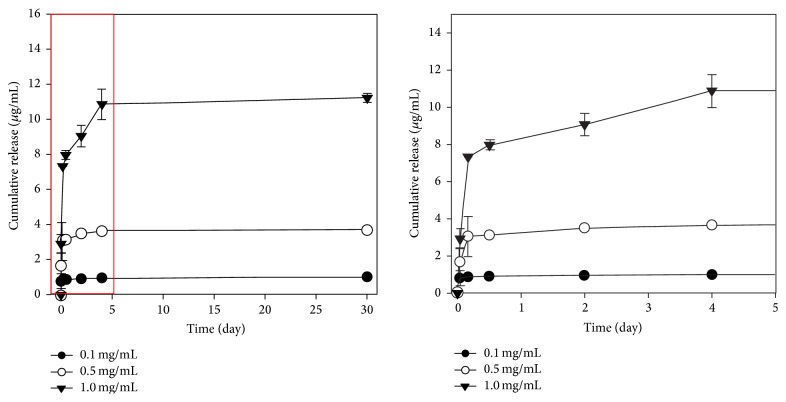
Release kinetics of rhBMP-2 in the three experimental groups. The 1.0 group showed a slower release tendency than the other groups, but all groups showed an initial burst release pattern.

As shown in Figure 4, 90% of rhBMP-2 was released from the 0.1 and 0.5 groups, and 70% was released from the 1.0 group over the first 6 hours in 1 mL PBS buffer (pH 7.4). After 4 days, ~100% of rhBMP-2 was released in all of the groups. The 1.0 group showed a slower release tendency. However, all groups exhibited an initial burst release pattern.

### 3.3. Clinical Findings and Stability Evaluation

All animals survived the surgical procedures, and the 24 implants healed uneventfully. None of the implants was lost or exposed during the healing period, and no clinical differences were detected among the four groups. Overall ISQ values were similar in all groups immediately after implantation. At week 8, ISQ values were higher than at baseline in all groups, and the 1.0 group showed higher ISQ values than the other groups. Increases in mean ISQ values were greater in the experimental groups than in the control group (Table 1).

### 3.4. Fluorescence Analysis

**Figure 5:**
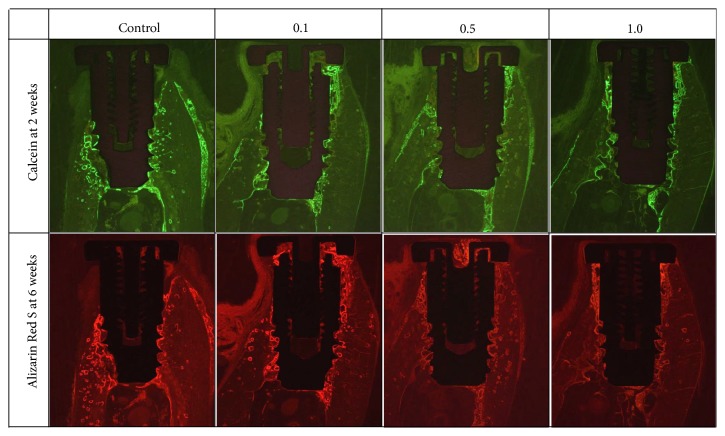
Fluorescent images obtained using a confocal laser microscope. In the 0.5 and 1.0 groups, massive bone remodeling was also observed in the early stage, and this was maintained in the late stage.

Specimens were allowed to heal for 8 weeks after implantation, and remodeled bone was observed within and adjacent to defect areas around implants in all groups. Fluorescent bone markers were used to indicate bone remodeling at certain times: Calcein (green) at 2 weeks and Alizarin Red S (red) at 6 weeks. At 2 and 6 weeks, the control group showed no significant differences in terms of bone formation. Unlike the other groups, active bone remodeling was not observed in the defect area in the control group. In the 0.1 group, massive bone remodeling was observed in the defect area during the early stage (~2 weeks after implantation), but no active bone formation was observed in buccal open defect areas. In the 0.5 and 1.0 groups, massive bone remodeling was also observed in the early stage, and this was maintained at the late stage (~6 weeks after implantation). Furthermore, a clear boundary was observed between areas of initial and late new bone formation (Figure 5).

### 3.5. Histological Analysis

Histological observations at 8 weeks after implantation showed bone formation and integration of implants with surrounding bone on the upper 3 mm straight portion of implant defect area in all four groups. Bone ingrowth from surrounding bone showed osseointegration in defect areas.

**Figure 6:**
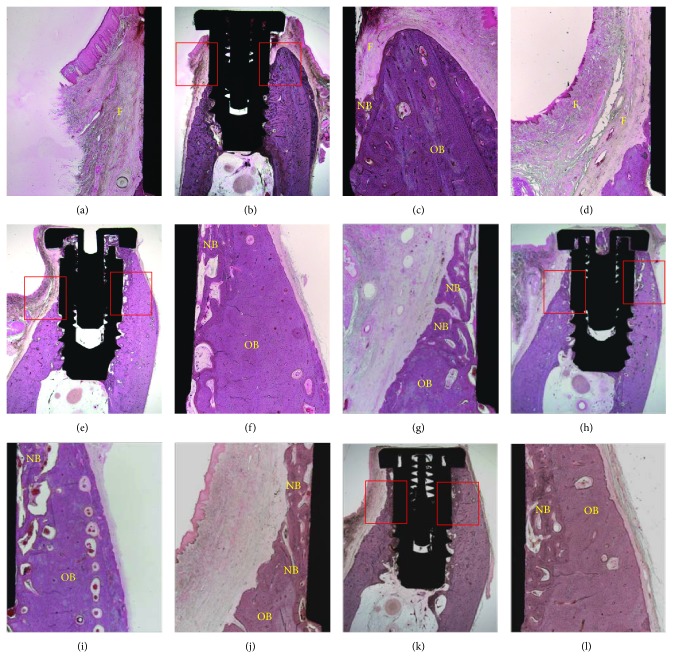
Images of histological specimens of each group obtained at 8 weeks: control group (a, b, and c), 0.1 group (d, e, and f), 0.5 group (g, h, and i), and 1.0 group (j, k, and l). Buccal side (a, d, g, and j). Lingual side (c, f, i, and l). F: fibrous tissue; NB: newly formed bone; OB: old bone (central original magnification ×12.5, left and right sides: ×40 original magnification).

The experiment groups (0.1, 0.5, and 1.0) showed clear boundaries between existing cortical bone and new bone growing in defect areas, whereas in the control group (Figures 6(a), 6(b), and 6(c)) no clear boundary was observed. In the 0.1 group (Figures 6(d), 6(e), and 6(f)), marginal bone in buccal defect area was not regenerated, but the lingual defect with a 1 mm gap was filled with new bone. Existing cortical bone showed a compact, lamellar appearance with osteons, whereas newly formed bone appeared less organized and less lamellar, and it was more consistent with woven bone. In the 0.5 (Figures 6(g), 6(h), and 6(i)) and 1.0 (Figures 6(j), 6(k), and 6(l)) groups, marginal bones were well regenerated in buccal and lingual areas, and new bone in defect areas was denser than in the other two groups (control and 0.1).

### 3.6. Histomorphometric Analysis

**Figure 7:**
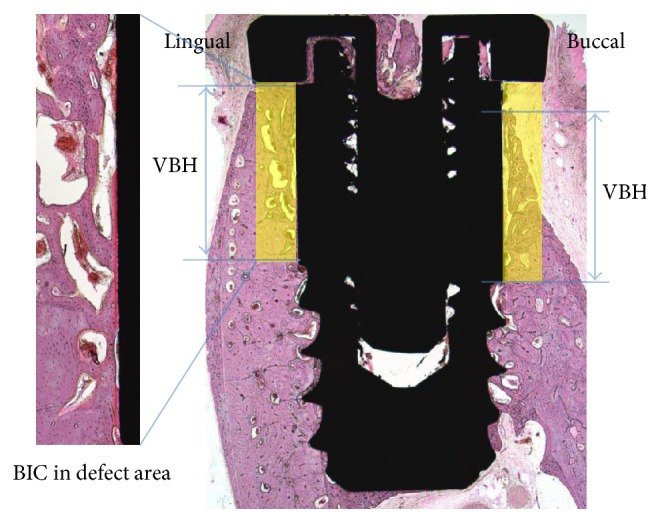
Parameters measured in histologic specimens: vertical bone height in the buccal open defect and lingual peri-implant defect areas (VBH, mm), bone-to-implant contact ratio for the upper 3 mm straight implant portions in buccal and lingual defect areas (BIC, red line, %), and bone volume in the buccal and lingual defect area (BV in yellow box, %).

Histomorphometric measurements are summarized in Figure 7 and Table 2. In all groups, mean values of vertical bone height in defect areas (VBH, mm), bone-to-implant contact ratio in defect areas (BIC, %), and bone volume in defect areas (BV, %) were greater in lingual defect areas than in buccal defect areas at 8 weeks after implant placement:

(1) *Vertical Bone Heights in Defect Areas (VBHs, mm)*. Mean VBHs of 1 mm lingual defect areas were similar in all groups. On the other hand, mean VBHs of buccal defect areas were higher in the 0.5 (1.88 ± 0.52) and 1.0 groups (2.06 ± 0.60) than in the control (−0.02 ± 0.62) and in the 0.1 groups (0.71 ± 0.62).
(2) *Bone-to-Implant Contact Ratios in Defect Areas (BIC, %)*. BIC is the most important factor for osseointegration. Mean BIC values in the 0.5 (24.47 ± 6.63) and 1.0 groups (18.42 ± 8.65) in buccal defect areas were higher than in the control (0.67 ± 1.15) and 0.1 groups (10.24 ± 10.99). Intergroup difference was not observed in lingual defect areas.
(3) *Bone Volume Percentages in Defect Areas (BV, %)*. In the investigation of new bone percentages on the surrounding 1 mm surface of the lingual defect area, mean BV (%) values of the experimental groups were higher than that of the control group. However, mean buccal BV (%) values of the 0.5 (33.67 ± 5.24) and 1.0 groups (35.67 ± 8.80) were greater than those of the 0.1 (13.30 ± 11.24) and control groups (2.77 ± 3.71).

## 4. Discussion

Based on the result of this present study in the buccal open defect area surrounding the SLA implant, coating with 0.5 and 1.0 mg/mL concentrations of rhBMP-2 was more effective, whereas in the lingual 1 mm coronal defect area rhBMP-2 exhibited no effect on promoting bone regeneration and osseointegration. The tested concentrations of rhBMP-2 (0.1, 0.5, and 1.0 mg/mL) were selected, based on an effective range of bone regeneration found in a previous study. Tatakis et al. [34] addressed that their radiographic and histometric analyses showed that none of 0.05, 0.1, and 0.2 mg/mL rhBMP-2 coated onto titanium implants showed meaningful differences in bone induction and osseointegration. According to Kim et al. [24] and Huh et al. [25, 33] studies, 0.75 mg/mL and 1.5 mg/mL rhBMP-2 coated anodized implants were successful at promoting bone formation and implant stability. Leknes et al. [29] and Wikesjö et al. [30] studies reported effective bone regeneration and osseointegration of anodized titanium porous oxide implants coated with 0.75, 1.5 mg/mL rhBMP-2, yet they also noted an undesirable implant displacement in the implant coated with the highest concentration (3.0 mg/mL). Compared to the other types of implants such as RMB and MgO, the SLA implants used in Lee et al.'s [32] study had the largest surface area, but they showed a low BIC value when 1.5 mg/mL rhBMP-2 was applied.

In the present study, SLA implants coated with different concentrations of rhBMP-2 were chosen and observed: like the nontreated group, 0.1 group showed an insignificant result, and both 0.5 and 1.0 groups whose concentrations were close to 0.75 mg/mL and lesser than 1.5 mg/mL seemed to be effective on bone regeneration and osseointegration. Thus, it was evident that SLA implants bring out a desirable outcome in a similar concentration range of rhBMP-2 like the other different implant surfaces. Through analyzing ISQ value, 0.5 and 1.0 mg/mL doses of rhBMP-2 were observed to contribute positive effects to implant stability. From the experimental group carried out at 0.5 mg/mL rhBMP-2, the highest BIC value was observed to be 3.212 ± 0.806 *μ*g. This BIC value was similar to that of TPO implants coated with 5 *μ*g of rhBMP-2 as Hall et al. [14] established in their study where, with different doses of rhBMP-2 (5, 10, or 20 *μ*g) on TPO implants in a rat ectopic model, coating amounts of 5 *μ*g of rhBMP-2 produced the most effective results in terms of bone formation, as confirmed by histometric analysis. Lee et al. [35] reported that, upon applying anodized implants with 4 different concentrations (0.1 mg/mL, 0.3 mg/mL, 0.5 mg/mL, and 1.0 mg/mL) of ErhBMP-2 on healthy alveolar bone, there was no improvement on BC and BIC values from all the experimental groups and that the highest BIC value was found at 0.3 mg/mL in dehiscence defect. However, this specific BIC value was lower than that of the nontreated implants or control group from Wikesjö et al.'s [30] study. In consideration of these experimental data, the effect of rhBMP-2 concentration was expected to show a wide range of deviations depending on size and morphology of bone defect around an implant. Therefore, in this study, local bone defect models that replicated various clinical conditions were schemed rather than commonly used supra-alveolar, peri-implant defect models [24, 25, 30, 33]. The defect model adopted in this study exhibited buccal open defect and lingual coronal defect areas around the upper 3 mm portion of implants. The design of this particular model was based on these following studies: animal study, bone healing at implant site with bone defects of varying dimension and configuration by Botticelli et al. [36] and a buccal dehiscence-type defect model by Schwarz et al. [37, 38] to examine bone regeneration around SLA implants. According to a study by Becker et al. [39], no significant differences were found among different surface treatment groups over 1 mm away from implant surfaces so that, in this study, the horizontal distance to margin was set as 1 mm away from implant surface.

From all the experimental groups, lower BV and BIC values were measured in buccal open defect compared with lingual coronal defect. rhBMP-2 coated onto implant surface is understood to be released in order to promote osteogenesis and increase osseointegration between implant and bone [30]. However, the limited new bone was formed in buccal open defect compared to lingual coronal defect. It is possible to reason that the buccal defect size was too large to form enough new bone by rhBMP-2 [36]. Within lingual coronal defect, the three different concentrations of rhBMP-2 displayed similar BV and BIC values and showed no significant difference from nontreated group (control). Based on these results, SLA implant without coating rhBMP-2 is expected to be successful at inducing bone regeneration and osseointegration when planted in coronal defect whose size is less than 1 mm. In this present study, SLA surfaces [8] were created by blasting with large grit particles (250–500 *μ*m) to create macroroughness and with an acid (HCl/H_2_SO_4_) to produce microroughness. Macroroughness of 18–23 *μ*m obtained by blasting with large grit enhances initial fixation and increases long-term physical stability, whereas microroughness of 2–4 *μ*m created by acid (HCl/H_2_SO_4_) enhances the osteoconductive process [7, 8, 31]. The upper 3 mm of SLA implants was designed to be straight so that the differences among treatments would be more apparent upon histological examination. The validity of SLA implant surface was reestablished by generating higher BV and BIC values, compared to those values of anodized implants coated with similar concentrations of rhBMP-2 to those used in this study, as addressed in the previous study [35].

In order to maximize the effect of rhBMP-2, it is crucial to standardize an ideal dose of rhBMP-2 for each of the different implant surfaces as well as to overcome a short half-life span of rhBMP-2. In this experiment, 0.5 and 1.0 groups that achieved successful bone regeneration and osseointegration showed 70~90% release of rhBMP-2 in the first 6 hours of implant plantation, and they exhibited an initial burst release pattern as 100% of rhBMP-2 was released completely in 4 days. The observed half-life span of rhBMP-2 in the present study was shorter than that in the previous study [25] where 0.75 mg/mL of rhBMP-2, coating anodized implant, released 36% of it on the first day of implant plantation and 67% of it in a week. For this reason, the upper 3 mm of the implant exposed to the bone defect area was designed to be straight so that a decreased surface area accounted for the limited absorption. However, a major limitation of this study occurred from not incorporating slow releasing system of rhBMP-2. To induce a constant and local release of rhBMP-2, these following carriers have been used: collagen gels [40], hyaluronic acid [41], fibrin gel [42], heparin [43–45], and so forth. Recent studies have found that rhBMP-2 immobilization on anodized titanium implant with heparin allows a constant release of rhBMP-2 for 28 days and is successful at improving osteoblast functions and bone formation [46, 47].

In this study, four beagle dogs were used for split-mouth design. Split-mouth design is an example of a randomization scheme on site level where two treatments are randomly assigned to sites in one of the two halves of the mouth. The attractiveness of the split-mouth design is the removal of much of the intersubject variability, thereby increasing the power of the study compared to the whole-mouth design. However, the limitation of this study was to make split-mouth design by small number of animals and implants. The experimental data collected from the four animals is not enough to provide a quantitative effect of the treatments. Since one animal is a statistical unit, it is hard to accept the presented statistical significance from a total of 2 observations/group.

Many studies have reported that rhBMP-2 at high concentration has harmful effects. Rosen [48] noted the formation of intrinsic BMP inhibitors at high BMP-2 concentrations, and Kaneko et al. [49] reported that osteoclasts were induced when BMP-2 concentrations were locally high. Huh et al. [25] mentioned that side effects, such as unsatisfactory new bone formation, resulted when a higher than appropriate concentration of rhBMP-2 was coated on implants, causing difficulties with regulating slow release. In the present study, the effect of slow release was minimal, and no ectopic bone formation caused by excessive rhBMP-2 was observed in any of the experimental groups. Thus, the concentrations of rhBMP-2 used in this study are acceptable for a treatment with SLA implant surface. The 0.5 and 1.0 mg/mL concentrations of rhBMP-2 were more effective at promoting bone regeneration and osseointegration in this pilot study. Based on these results and limitations of our study, we have planned to carry out further split-mouth designed animal study with various implant surfaces, more subdivided rhBMP-2 concentrations, defect models that better replicate clinical conditions, and application of slow releasing system. In this study, we focused on effectiveness of different concentration of rhBMP-2 onto SLA implant surfaces on bone regeneration and osseointegration in a bone defect model. The present study manifests the following: in the 1 mm coronal bone defect area surrounding the implant, bone mass and density were increased merely by the SLA implant surface, but in the open bone defect area, 0.5 and 1.0 mg/mL concentrations of rhBMP-2 were more effective at promoting bone regeneration and osseointegration.

## Figures and Tables

**Table 1:** Implant stability quotient (ISQ) values at 8 weeks after implantation.

Group	At surgery	At 8 weeks	ISQ change
Control	46.83 ± 3.97	60.17 ± 3.25	13.33 ± 5.43
0.1	48.00 ± 4.00	64.83 ± 3.19	16.83 ± 4.67
0.5	49.83 ± 5.95	71.67 ± 6.15	19.83 ± 9.56
1.0	52.67 ± 7.61	72.00 ± 2.68	18.17 ± 6.94

At surgery: ISQ value at surgery.

At week 8: ISQ value 8 weeks after implantation.

**Table 2:** Means (±SDs) of VBH (mm), BIC (%), and BV (%) of the upper 3 mm straight portion of implant in buccal and lingual defect areas at 8 weeks after implantation.

Group	VBH (mm)		BIC (%)		BV (%)	
	Buccal	Lingual	Buccal	Lingual	Buccal	Lingual
Control	−0.02 ± 0.62	2.43 ± 0.29	0.67 ± 1.15	23.37 ± 7.08	2.77 ± 3.71	46.50 ± 11.36
0.1	0.71 ± 0.62	2.70 ± 0.34	10.24 ± 10.99	26.50 ± 10.97	13.30 ± 11.24	60.50 ± 14.60
0.5	1.88 ± 0.52	2.80 ± 0.23	24.47 ± 6.63	35.45 ± 7.16	33.67 ± 5.24	66.17 ± 13.00
1.0	2.06 ± 0.60	2.88 ± 0.16	18.42 ± 8.65	33.43 ± 11.39	35.67 ± 8.80	65.00 ± 15.76

VBH: vertical bone height (mm); BIC: bone-to-implant contact in the defect area (%); BV: bone volume in defect area.
